# Therapeutic repair for spinal cord injury: combinatory approaches to address a multifaceted problem

**DOI:** 10.15252/emmm.201911505

**Published:** 2020-02-24

**Authors:** Jarred M Griffin, Frank Bradke

**Affiliations:** ^1^ Laboratory for Axonal Growth and Regeneration German Centre for Neurodegenerative Diseases (DZNE) Bonn Germany

**Keywords:** axon regeneration, clinical trials, combination treatments, reproducibility, spinal cord injury, Neuroscience, Chemical Biology

## Abstract

The recent years saw the advent of promising preclinical strategies that combat the devastating effects of a spinal cord injury (SCI) that are progressing towards clinical trials. However, individually, these treatments produce only modest levels of recovery in animal models of SCI that could hamper their implementation into therapeutic strategies in spinal cord injured humans. Combinational strategies have demonstrated greater beneficial outcomes than their individual components alone by addressing multiple aspects of SCI pathology. Clinical trial designs in the future will eventually also need to align with this notion. The scenario will become increasingly complex as this happens and conversations between basic researchers and clinicians are required to ensure accurate study designs and functional readouts.

## Graphical abstract

This review is comprehensive and timely, and provides to the readers a balanced view of research and clinical findings in spinal cord injury.

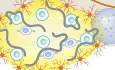

**Glossary**
Allodynia: A type of neuropathic pain that is the result of increased sensitivity to otherwise innocuous stimuli
Anterograde tracing: This is a research method which is used to trace axonal projections in the direction of their cell bodies towards their point of termination. The complimentary technique is known as retrograde tracing
Autologous (transplant): A transplant is autologous if the recipient also serves as the donor
Basso, Beattie and Bresnahan (BBB) locomotor rating scale: A 21‐point behavioural analysis scale that is used in spinal cord injury research to assess limb motor skills over a wide range of injury severities
Chondroitin sulphate proteoglycans (CSPGs): Components of the extracellular matrix and are composed of a protein core covalently linked to chondroitin sulphate glycosaminoglycan sidechains. CSPGs are known to inhibit axon regeneration
Chondroitinase ABC (ChABC): Chondroitinase ABC is a bacterial enzyme that degrades chondroitin sulphate
Conditioning lesion: These involve lesioning the peripheral branch of adult sensory dorsal root ganglion neurons prior to a central lesion. This increases the regenerative capacity of the central branch of DRG neurons
Contusion: An injury caused by blunt trauma to the spinal cord
Corticospinal tract (CST): One of the most important descending tracts. It controls primary motor activity from the motor cortex
Dorsal root ganglion (DRG): A cluster of neurons in dorsal spinal root of a spinal nerve. DRG neurons are pseudo‐unipolar and have a central and peripheral branch
Excitotoxicity: A pathological process by which neurons are damaged or killed through overstimulation of excitatory neurotransmitter receptors
Functional electrical stimulation (FES): FES involves electrophysiological stimulation of spinal cord or peripheral nerves or muscle
Growth cone: Is the large actin supported extension at the tip of a developing or regenerating neurite/axon
Induced pluripotent stem cells (iPSCs): Are pluripotent stem cells that are generated directly from adult cells through the expression of specific transcription factors
Kinematic analysis: The process of measuring the movement (kinematic) quantities used to describe motion
Myelin‐associated inhibitors: Components or CNS myelin that are known to be inhibitors of axon growth and regeneration. The three classic myelin‐associated inhibitors are Nogo‐A, myelin‐associated glycoprotein and oligodendrocyte myelin glycoprotein
Neuroplasticity: Refers to the adaptive changes in neurological function. This can be the result of anatomical or physiological changes
Neuroprotection: Refers to the relative preservation of neural tissue during ongoing secondary damage
Neurorehabilitation: Physical therapy that aims to aid recovery of nervous system injury and/or improve compensatory functions
Neurotrophins: A family of regulatory factors that mediate the survival, differentiation and growth of neurons
Phase I clinical trial: A clinical trial that includes a small number of human participants to determine the safety of a new drug or invasive medical device; allows the determination of drug dosage or toxicity limits
Phase II clinical trial: A clinical trial that includes a larger number of human participants than a Phase I trial and is intended to evaluate the efficacy of a treatment; side effects are also monitored
Propriospinal neuron: These are neurons contained entirely within the spinal cord that interconnect various levels of the cord
Reticulospinal tract: A descending tract that descends from the reticular formation and is primarily responsible for locomotion and postural control
Retraction bulb: Dystrophic axonal structures which are a hallmark of a failed growth response
Transection: A surgical injury created by a fine transverse cut of the spinal cord

## Introduction

Through silver staining neurons, Ramon y Cajal discovered that peripheral nervous system (PNS) neurons regenerate after injury, contrasting the minor regenerative response of central spinal cord neurons (Ramon y Cajal, 1928). This then prompted the question: Do central nervous system (CNS) neurons lack the intrinsic capability of regeneration or are there extrinsic factors that influence this dichotomous observation? In actuality, both of these factors come into play when spinal cord neurons are tasked with regenerating after axotomy. Several families of molecules present in the extracellular matrix (ECM) prevent axon growth including chondroitin sulphate proteoglycans (CSPGs), myelin‐associated molecules, ephrins and semaphorins (Miranda *et* *al,* 1999; Chen *et* *al,* 2000; Willson *et* *al,* 2002; Silver & Miller, 2004; Geoffroy & Zheng, 2014; Worzfeld & Offermanns, 2014). Yet even when provided with a growth‐permissive environment, central neurons regenerate feebly compared to their peripheral or immature CNS counterparts, indicating that they also have intrinsic growth limiting factors (Hilton & Bradke, 2017). On the brighter side and nearly 100 years on from Cajal's statement that “in adult centres, the nerve paths are something fixed, ended, immutable; everything may die, nothing may be regenerated” (Ramon‐Cueto *et al,* 1998), we now know this statement to be not entirely true. Many research groups have reported axon regeneration and functional recovery after experimental spinal cord injury (SCI) following a variety of treatments (Thuret *et* *al,* 2006). While this is certainly a feat, many of the experimental approaches are problematic for clinical translation. For those that are, it is also becoming increasingly apparent that addressing singular aspects of the problem won't facilitate successful and functional regeneration after SCI in humans. Conversely, it will likely require the combination of various treatment strategies that address the variety of problems that result after SCI. Many attempts have been made, to varying degrees of success, that combine tissue replacement, removal of inhibitory molecules, supplying neurotrophic factors, manipulation of pro‐regenerative neuronal signalling pathways and neurorehabilitation. This review will provide an update to the many therapeutic interventions after SCI and then will focus on the attempted combinatory approaches, how they could be improved and the road to their clinical translation.

## Therapeutic interventions

**Figure 1:**
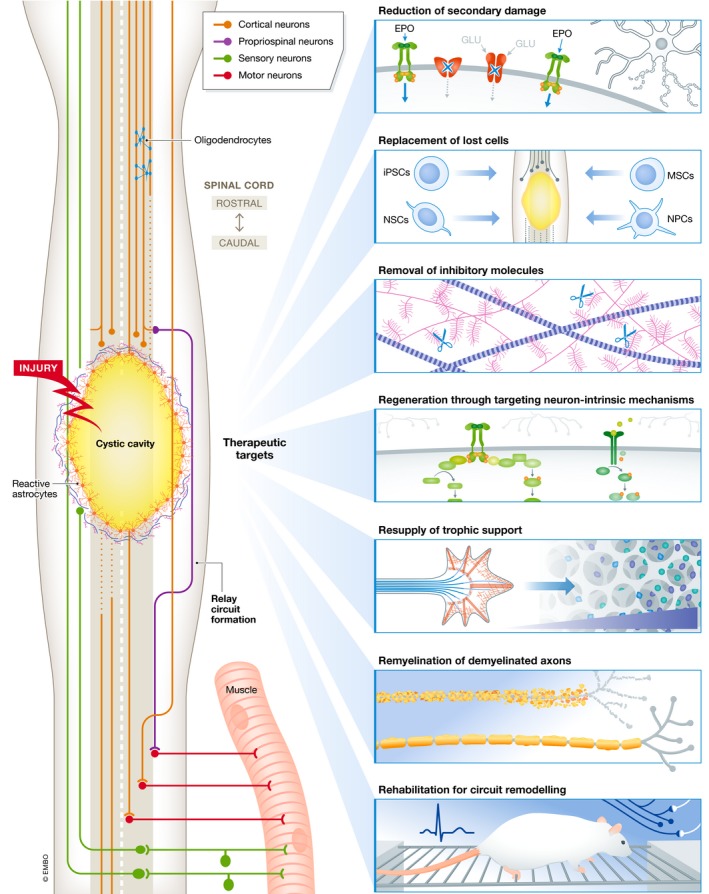
The seven targets for therapeutic interventions following spinal cord injury A horizontal plane view through a region of thoracic spinal cord injury depicting some of the features of the pathology. SCI leads to immediate and continued death of neural alongside disruption of descending and ascending fibres. Seven therapeutic targets are present which can improve functional recovery after SCI: neuroprotective strategies to limit ongoing secondary damage resulting in spared tissue; tissue and cellular transplants to replace lost cells and may provide trophic or growth‐permissive environments; removal of inhibitory factors such as CSPGs to allow for enhanced axonal growth; targeting neuron‐intrinsic mechanisms to enhance intrinsic regenerative response which could then be directed through the resupply of trophic support; and remyelination of demyelinated axons may improve axonal conduction. Finally, rehabilitation to function in circuit remodelling and strengthens beneficial connections.

SCI induces complex processes. SCI first leads to death of cells in the CNS, including neurons, astrocytes, microglia, oligodendrocytes and endothelial cells. In particular, the damage to long axonal projections leads to interruption of descending and ascending pathways that transmit information between the brain and the rest of the body. Secondary damage from vascular changes, acute injury signalling, neuroinflammation, excitotoxicity, demyelination, degeneration, astrogliosis and ECM remodelling exacerbates the initial pathology (Hilton *et* *al,* 2017; Bradbury & Burnside, 2019). This unfolds as a temporal cascade of complex biological processes that can last months to years after the injury (Buss *et* *al,* 2004; Norenberg *et* *al,* 2004; Donnelly & Popovich, 2008). Some degree of spontaneous recovery is observed in experimental animal models and to a lesser extent in humans (Curt *et* *al,* 2008; Hilton *et* *al,* 2016). However, endogenous repair mechanisms are minor and recovery remains incomplete (Fawcett *et* *al,* 2007; Courtine *et* *al,* 2008). Based on the pathologies that result from SCI, researchers have identified several targets for the development of potential therapeutic interventions. These can be referred to as the “7 R's” (Fig 1).

1 **Reduction** of secondary damage (neuroprotection).
2 **Replacement** of cells lost to primary and secondary damage.
3 **Removal** of inhibitory molecules.
4 **Regeneration** to enhance the spontaneous reparative and regenerative responses.
5 **Resupply** of neurotrophic support to improve neuronal survival and direct axonal growth.
6 **Remyelination** of regenerated, replaced or spared (demyelinated) axons.
7 **Rehabilitation** strategies to induce neuroplasticity and/or to shape neuronal connections.

We will discuss these targets and their respective interventions in the following sections.

### Reduction: neuroprotection

Substantial efforts have been made to limit secondary damage. Pharmacological agents that suppress the immune system or inhibit key signalling pathways involved in inflammation were the first major strategies applied to patients. These include the non‐steroidal anti‐inflammatory drugs (NSAIDs), minocycline, cyclosporine A and the corticosteroid methylprednisolone (Badhiwala *et* *al,* 2018). The use of methylprednisolone remains controversial since clinical trials revealed that it may have no effect or even lead to complications (Bowers *et* *al,* 2016). Preclinically, indomethacin (an NSAID) led to tissue sparing and slight functional recovery (Simpson *et* *al,* 1991), while minocycline treatment reduced oligodendrocyte as well as neuronal death that improved outcome after cervical spinal cord injury in rats (Stirling *et* *al,* 2004). However, preclinical reassessment of indomethacin suggested treatment may be harmful in human SCI, and a phase II clinical trial of minocycline for acute SCI did not report beneficial effects (Guven *et* *al,* 1999; Casha *et* *al,* 2012). Similar conflicting results have been reported for the immune suppressant cyclosporine A (Chen *et* *al,* 2018). Since neuroinflammation has both beneficial and detrimental effects, broad‐spectrum suppression of inflammation may not be efficacious. Neuroprotection can also be achieved through preventing glutamate excitotoxicity by blockade of NMDA receptors by magnesium (Ditor *et* *al,* 2007) or gacyclidine (Feldblum *et* *al,* 2000), blockade of tetrodotoxin‐sensitive sodium channels using riluzole (Satkunendrarajah *et* *al,* 2016), preventing apoptosis using erythropoietin (Baptiste & Fehlings, 2006), inhibition of connexin hemichannels using a mimetic peptide (Mao *et* *al,* 2017), mild to moderate hypothermia (Dietrich *et* *al,* 2009) and many more strategies (Baptiste & Fehlings, 2006; Thuret *et al,* 2006). These more discrete manipulations of secondary processes may prove to have greater therapeutic benefit than the broad approaches. Regardless, neuroprotective agents have failed to translate to the clinic. Stroke has a similar neuropathology to SCI, and huge efforts, particularly in the 1990s, were made to develop neuroprotective treatments for stroke. In such attempts, over 1,000 potential therapeutic agents, leading to nearly 200 clinical trials, have resulted in no successful treatments (Minnerup *et* *al,* 2012). Worryingly, clinical trials assessing neuroprotective agents for SCI may also reflect this trend. Overall, the preclinical and clinical progression of neuroprotective strategies has often stalled, and the mechanisms by which neuroprotection is conferred is not well understood. Improvements in our understanding as to the mechanisms which underpin the benefits of neuroprotective strategies could better inform their application.

### Replacement: cellular transplantation

Cell transplantation for the aim of replacing lost cells has multiple historic origins stretching back to experiments conducted in the laboratory of Ramon y Cajal (Ramon‐Cueto *et al,* 1998). The seminal works of Anders Björklund and colleagues showed that foetal tissue can promote CNS repair by replacing lost cell types in models of Huntington's and Parkinson's disease (Bjorklund & Lindvall, 2000). This led to the discovery that transplantation of foetal spinal cord tissue into the injured spinal cord results in successful graft survival, differentiation and integration into the host tissue (Bregman *et* *al,* 1993). Likewise, experiments by Richard, David and Aguayo revealed that peripheral nerve grafts could provide a permissive conduit for regenerating CNS axons (Richardson *et* *al,* 1980, 1984; David & Aguayo, 1981; Benfey & Aguayo, 1982), which has since been thoroughly studied (Cote *et* *al,* 2011a). These studies provided critical evidence that CNS axons are capable of regenerating. The grafts support a variety of neuronal types including propriospinal neurons and often neurons from the brain stem (Cote *et al,* 2011a). It appears that only injured axons regenerate through the grafts and not sprouts from non‐injured axons (Friedman & Aguayo, 1985). Despite these promising findings, there are few studies using PNGs alone which correlate this axonal growth to behavioural or recovery of axonal conduction. The likely reason for this is the remaining challenge that CNS and PNS neurons fail to extend beyond the distal graft–host interface where they encounter the inhibitory environment of the CNS. For example, lack of axon extension and functional improvements were observed in primates after spinal hemisection and PNG transplantation (Levi *et* *al,* 2002).

Cell‐based transplantations have largely superseded nerve grafts for several reasons: they can be injected into the spinal cord to fill the lesion site, they are less likely to cause further damage compared to a nerve graft, and cells can be genetically modified *ex vivo* to secrete specific growth factors (Assinck *et* *al,* 2017). The mechanisms behind which cell transplantation confers therapeutic benefit are often multifactorial. This includes direct replacement of damaged neural cells, neuroprotection of the host cells, promoting axon regeneration and synapse formation, and/or promoting myelination of damaged or newly formed axons (Assinck *et al,* 2017). A variety of cells have been used for SCI transplantation, including mesenchymal stem cells (MSCs), neural progenitor cells (NPCs), Schwann cells, olfactory ensheathing cells (OECs) and induced pluripotent stem cells (iPSCs; Assinck *et al,* 2017). Each has advantages and disadvantages when compared to one another.

MSCs and NPCs are stem cells, typically harvested from embryonic or foetal tissue, and can differentiate into neurons or glia *in vitro* (Liu *et* *al,* 2000; Billon *et* *al,* 2006). Neurons obtained from *in vitro* differentiation can survive and integrate into the injured rat spinal cord (Deshpande *et* *al,* 2006). Whether such differentiation occurs following *in vivo* transplantation has been less clear in some circumstances considering early investigations reported that majority of transplanted cells remain progenitor‐like or differentiate into glial cells (Vallieres & Sawchenko, 2003; Karimi‐Abdolrezaee *et* *al,* 2006). It is now generally accepted that MSCs do not form neurons *in vivo* (Lu *et* *al,* 2004). NPCs, on the other hand, were recently shown to extend “hundreds of thousands of axons into the spinal cord” (Rosenzweig *et* *al,* 2018), and injured motor and sensory axons regenerated into appropriate domains of NPC grafts (Dulin *et* *al,* 2018). Furthermore, these NPC grafts form host–graft synaptic network formation in patterns paralleling the normal spinal cord (preprint: Ceto *et* *al,* 2019). Aside from direct tissue replacement, both MSCs and NPCs confer permissive substrates for growth whereby injured host axons grow into the cellular grafts (Hofstetter *et* *al,* 2002; Lu *et* *al,* 2003; Ankeny *et* *al,* 2004). Injured axons grow into an NPC transplant, mediated by the secretion of various neurotrophic factors from the transplant itself (Lu *et al,* 2003). Similar to that observed in PNS grafts, regenerating host axons typically terminate their growth at the border of the transplant (Ruff *et* *al,* 2012; Assinck *et al,* 2017). A final consideration is that NPCs may also facilitate growth via paracrine actions. For example, modulation of neuroinflammatory processes may also contribute to the beneficial effects NPCs (Kokaia *et* *al,* 2012).

Schwann cells and OECs are terminally differentiated, myelinating and regeneration promoting cells found in the PNS and the olfactory system, respectively. Like other transplantation studies, both Schwann cells and OECs confer structural and trophic support (Bunge & Pearse, 2003; Barnett & Riddell, 2007). Schwann cells transplanted into the damaged spinal cord of rodents reduce cavitation and promote regeneration of both ascending and descending axons into the graft, and axons became myelinated and are evidenced to be electrophysiologically active (Xu *et* *al,* 1995b, 1997; Pinzon *et* *al,* 2001; Takami *et* *al,* 2002). However, similar to MSC and NPC transplants, in these studies, regenerating axons failed to leave the graft distally to reinnervate the host tissue. Recovery of limb functions was reported by some (Takami *et al,* 2002) but not all publications (Pearse *et* *al,* 2004a). Similar observations have been witnessed for OECs. After injection of OECs into a cervical unilateral lesion site of the corticospinal tract (CST) in rodents, anterograde tracing of lesioned hindlimb CST axons revealed extensive regeneration through the graft and integration with the CST beyond the injury site (Li *et* *al,* 1997). Later, it was shown that this intervention could improve respiratory function and climbing ability of the rats (Li *et al,* 2003). However, it was later challenged whether OECs promote CST regeneration (Lu *et* *al,* 2006). Other studies, including veterinary trials, have also shown the ability of OECs to promote robust regeneration of some spinal pathways even beyond the graft to enhance functional recovery (Ramon‐Cueto *et* *al,* 1998; Lu *et* *al,* 2002; Li *et* *al,* 2003; Jeffery *et* *al,* 2005; Toft *et* *al,* 2007). The benefits of Schwann cells and OECs can be potentiated when delivered together. For example, co‐delivery of the two cell types potentiates long‐distance axonal regeneration through and around guidance channels compared to what is achieved by either the cell type alone (Ramon‐Cueto *et al,* 1998).

iPSCs may circumvent the ethical issues associated with embryonic or foetal tissue use. NPCs generated from iPSCs have demonstrated beneficial effects after transplantation for animal models of SCI (Nagoshi & Okano, 2018). It remains unclear whether meaningful differentiation into neurons occurs or whether they play a supportive role. As is the case with other pluripotent cells, a critical safety issue for the use of iPSCs is the risk of tumorigenicity. Though promisingly, in marmosets, transplanted iPSC‐NPCs at the subacute phase predominantly differentiated into neurons around the lesion site without tumorigenicity and promoted axonal regrowth and angiogenesis, and preserved myelination area (Kobayashi *et* *al,* 2012). Furthermore, one strategy to decrease the risk of tumorigenicity is the addition of gamma‐secretase inhibitors, which removes tumour‐initiating cells and promotes functional recovery in the subacute and chronic phases of SCI in preclinical studies (Okubo *et* *al,* 2016, 2018).

Overall, preclinical SCI studies have demonstrated that various cellular transplants are efficacious through many mechanisms. Considering the limitations listed above, there remains much experimentation. The full potential of any cell type for transplantation will likely require the combination with other synergistic therapies.

### Removal: targeting chondroitin sulphate proteoglycans

Removal or disruption of CSPGs can be achieved by inhibiting their synthesis, enzymatic degradation, antibody neutralisation or pharmacological targeting of effector molecules (Bradbury & Burnside *et* *al.,* 2017). For example, xylosyltransferase‐1 (XT‐1) is crucial for the biosynthesis of glycosaminoglycan (GAG) chains of CPSGs. Reduction of XT‐1 expression through using a DNA enzyme for catalytic degradation of XT‐1 mRNA strongly reduced CSPG‐GAGs and allowed for axons from microtransplanted dorsal root ganglions (DRGs) to grow around a dorsal column lesion in rats (Grimpe & Silver, 2004). In a similar study, mRNA‐mediated knockdown of XT‐1 after dorsal column transection in rats significantly reduced proteoglycan expression and increased the length and density of ascending axons through a peripheral nerve graft (Hurtado *et* *al,* 2008). This was later replicated in a model of spinal cord contusion where increases in serotonergic innervation caudal to the injury correlated with a reduction in errors during a horizontal ladder test (Oudega *et* *al,* 2012).

Enzymatic modification of CSPGs is most commonly achieved with the enzyme chondroitinase ABC (ChABC). Derived from the bacteria *Proteus vulgaris,* it catalyses the degradation of the glycosidic bonds between CS‐GAGs of CSPGs, liberating them from the CSPG core protein (Prabhakar *et* *al,* 2005a, 2005b). Intrathecal infusion of ChABC for 10 days into rats following C4 dorsal column crush lesion degraded CSPGs, increased CST‐axonal regeneration and improved functional scores on several behavioural tests (Bradbury *et* *al,* 2002). Using anterograde tracing and immunohistochemical markers for neuroplasticity, ChABC treatment promoted sprouting of intact and injured spinal systems and formed relay networks after SCI (Barritt *et* *al,* 2006). The beneficial effects of ChABC delivery have been replicated by many independent laboratories in a variety of injury models (Bradbury & Carter, 2011). The positive results of using ChABC in rodents have been further confirmed in larger animals. In adult cats following thoracic hemisection, for example, ChABC enhanced functional recovery of skilled locomotion and kinematic measurements of hindlimb function (Tester & Howland, 2008). Importantly, in a recent SCI clinical trial in dogs, ChABC resulted in a seemingly modest, yet significantly improved forelimb–hindlimb coordination and early improvements to bladder compliance with no evidence of long‐term adverse effects (Hu *et* *al,* 2018). While average group differences are small, 10% of the treated dogs recovered independent ambulation which may represent an estimate of the “true” population effect is in such a large and heterogeneous sample size of 60 dogs (Moon & Bradbury, 2018). Cervical hemisection in rhesus monkeys and intrathecal administration 4 weeks later resulted in increased CST axon growth and formation of synapses from CST axons caudal to the lesion (Rosenzweig *et* *al,* 2019). This correlated with improved hand function compared to vehicle‐treated controls. Increasing neuroplasticity may even have long‐lasting effects. A single injection of ChABC into the phrenic motor pool following complete cervical hemidiaphragm paralysis in rats resulted in robust patterned respiratory recovery for more than 1.5 years (Warren *et* *al,* 2018). As such, through unmasking of the neuroplasticity that develops after injury, ChABC treatment can ensure rapid and robust functional recovery after a near lifetime of paralysis in rats.

The continuous production of CSPGs after injury may require chronic expression of ChABC. For this reason, a mammalian‐compatible ChABC gene was engineered by modifying N‐glycosylation sites to allow for secretion of ChABC from eukaryotic cells (Muir *et* *al,* 2010). This also avoids tissue damage of repeated administration and addresses the possible risks for immune recognition of the bacterial enzyme. Lentiviral vector (LV) gene delivery was used to achieve long‐term expression of this “mammalianised” ChABC (mChABC) gene in the contused rat spinal cord which resulted in large‐scale CSPG degradation and improved behavioural scores following thoracic injury (Bartus *et* *al,* 2014). Recently, these findings were replicated in the functional restoration of upper limb function and the strategy further developed to control gene expression (James *et* *al,* 2015). An immune‐evasive, doxycycline Tet‐on inducible LV‐mChABC vector enabled temporal control over ChABC expression (Burnside *et* *al,* 2018). Such regulatable gene therapy approach could be a viable candidate for clinical translation.

There are endogenous proteins which also may affect matrix composition that could be therapeutically harnessed. These include matrix metalloproteinases (MMPs), a disintegrin and metalloproteases (ADAMs) and a disintegrin and metalloproteinase with thrombospondin motifs (ADAMTSs; Troeberg & Nagase, 2012). MMPs, ADAMs and ADAMTSs have been implicated to play a beneficial role in CNS injury by degrading CSPGs (Burnside & Bradbury, 2014). For example, knockout of MMP‐9 in mice caused worsened motor deficit after traumatic brain injury (Wang *et* *al,* 2000). ADAMs and ADAMTSs are of particular interest as they display CSPG‐specific substrate recognition. ADAMTS4 infusion intrathecally in the contused rat spinal cord resulted in a significant improvement in the Basso, Beattie and Bresnahan (BBB) locomotor rating scale score that was comparable to ChABC infusion (Tauchi *et* *al,* 2012). More recently, an astrocyte‐selective AAV‐ADAMTS4 gene therapy resulted in CSPG degradation, decreased lesion size, increased CST axon regeneration, increased caudal serotonergic inputs and significantly improved BBB and error ladder scores following thoracic contusion in rats (Griffin *et* *al,* 2020). Given the safety profile of AAV viruses (Hudry & Vandenberghe, 2019), this gene therapy could potentially represent a safer alternative to LV‐ChABC gene therapy. Ultimately, removal of CSPGs has proven to be consistently advantageous for nearly two decades and we anticipate the next steps taken towards clinical translation of CSPG‐targeting therapies.

### Removal: targeting myelin‐associated inhibitors

Independent laboratories have reported CNS axon growth and recovery of limb function following the use of anti‐Nogo‐A antibodies (Chen *et al,* 2000; GrandPre *et* *al,* 2000). In non‐human primates, anti‐Nogo‐A antibody therapies promoted the growth of CST tract axons after unilateral dorsal hemisection and regain of fine hand control (Freund *et* *al,* 2009). Interestingly, the co‐delivery of anti‐Nogo‐A antibodies and ChABC is more effective than single treatments at enhancing functional recovery after SCI indicating that removal of multiple inhibitory factors is advantageous (Zhao *et* *al,* 2013). Other therapeutic targeting of Nogo includes a soluble Nogo‐66 receptor protein (NgR (310) ecto‐FC) that complexes with Nogo‐A. NgR (310) ecto‐FC enhances CST and raphespinal axon growth after dorsal hemisection, and after complete transection in rats significantly enhances the number myelinated fibres in the bridge and increases the area of the bridging tissue between the cord stumps (Li *et* *al,* 2004; Guo *et* *al,* 2012). The Nogo receptor antagonist NEP1‐40 originally promoted CST and serotonergic fibre growth following thoracic dorsal hemisection in rats with striking results (GrandPre *et* *al,* 2002). The promising results obtained from the last three decades of preclinical research investigating Nogo‐A and anti‐Nogo‐A antibodies led to the first‐in‐man intrathecal application of anti‐Nogo‐A antibodies in a phase I human clinical trial of cute spinal cord injury (Kucher *et* *al,* 2018), and further trials regarding NOGO intervention are currently ongoing (NCT03935321, NCT03989440).

### Regeneration: targeting neuronal intrinsic mechanisms

Intrinsic growth capacity of central neurons declines with age as the neuron differentiates for synaptic functions (Hilton & Bradke, 2017). This is a major reason why they mount a minor regenerative response following an injury. Through studying developing immature neurons and regenerative‐competent peripheral neurons we know that for an axon to regenerate, many diverse and coordinated intracellular mechanisms are required. These include cytoskeleton dynamics, axonal transport and trafficking, signalling and transcription of regenerative programmes, and epigenetic modifications (Curcio & Bradke, 2018; Fawcett & Verhaagen, 2018).

Reactivation of an intrinsic growth programme can be accomplished through conditional lesioning which involves transecting the peripheral branch of adult sensory DRG neurons prior to a central lesion (Richardson & Issa, 1984). Studies correlated cyclic adenosine monophosphate (cAMP) levels in DRG neurons to growth capacity through induction of important pro‐regenerative transcription factors (Qiu *et* *al,* 2002). Local administration of cAMP to DRGs can, at least in part, mimic the conditioning lesions (Cai *et* *al,* 2001; Neumann *et* *al,* 2002; Qiu *et al,* 2002). Rolipram prevents the hydrolysis of cAMP and sustains elevated levels of cAMP that mimic the effects of a conditioning lesion effect to some extent (Nikulina *et* *al,* 2004; Pearse *et* *al,* 2004b). cAMP‐mediated gene transcription alone only partially recapitulates the conditioning lesion effect (Blesch *et* *al,* 2012), indicating that there are opportunities remaining to discover therapeutic interventions that do so.

Genetic manipulation experiments, including deletion of PTEN or GSK3β, or overexpression of STAT3, KLFs or SOX11, have exemplified that these manipulations to aspects of regenerative programmes can produce a modest regenerative response (Curcio & Bradke, 2018). Converting these interventions into clinically useable alternatives such as pharmacological agents or viral vector gene therapies of these could be a promising avenue (Blackmore *et* *al,* 2012; Zukor *et* *al,* 2013; Wang *et* *al,* 2015). Another strategy is to modulate intracellular signalling through modulation of GTPases. This has been achieved through RhoA‐GTPase inhibitors, C3‐peptides, C3‐ADP‐ribosyltransferase, siRNA and ROCK inhibitors (Wu & Xu, 2016). Blocking of Rho activation using Cethrin (BA‐210; SPRING‐VX‐210) has shown promise in preclinical and multicentre phase I/IIa clinical trials; some patients with one dose reported improvements in an ASIA grade (Fehlings *et* *al,* 2011). A further phase 2b/3 study determining efficacy and safety was initiated for patients with acute traumatic cervical SCI (Fehlings *et* *al,* 2018), although regrettably it was discontinued shortly after due to lack of efficacy after an interim analysis (Taylor WEBARTICLE, 2018). Similarly, inhibition of the downstream effector Rho kinase (ROCK) by Y27632 and Fasudil in mouse models of SCI resulted in similarly inefficacious effects of RhoA inhibition when delivered acutely (Borisoff *et* *al,* 2003; Chan *et* *al,* 2005; Watzlawick *et* *al,* 2014). While still an intriguing target, one key reason for the unsuccessful trial is that we still understand relatively little about the mode of action of Rho blockade, both for neuronal and non‐neuronal cells. We have only recently gained a deeper understanding of the physiological role of RhoA in axon growth during development and its downstream effectors. RhoA restrains axon initiation through RhoA/myosin‐II‐dependent actin arcs that restrict microtubule advancement in the growth cone (Dupraz *et* *al,* 2019). Future research needs to further disentangle the physiological role of RhoA in axon regeneration and how RhoA‐dependent mechanisms in glial cells might affect axon regeneration.

The cytoskeleton plays an important role in axonal growth in development and regeneration. Microtubules underlie the formation of retraction bulbs and are a major contributor to the failure of axonal regeneration; stabilisation of microtubules specifies growth cone formation and protrusion (Erturk *et* *al,* 2007; Witte *et* *al,* 2008). Stabilisation of microtubules also prevents polarisation and migration of scar‐forming fibroblasts which is associated with decreased deposition on CSPGs (Ruschel *et* *al,* 2015). Independent laboratories have shown that systemic administration of low doses of the microtubule‐stabilising drugs epothilone B or D after SCI results in adequate CNS penetration and distribution, reduced deposition of inhibitory proteoglycans, reduced axon die‐back, induced axonal growth and improved motor functions after SCI (Ruschel *et al,* 2015; Ruschel & Bradke, 2018; Sandner *et* *al,* 2018). Considering these drugs are FDA‐approved, easily administered and address both intrinsic and extrinsic determinants of axon regeneration, they represent a promising therapeutic candidate. Further evidence that implicates microtubule dynamics as important regulators of axon development and regeneration comes from studies investigating collapsin response mediator protein 2 (CRMP2). This protein stabilises microtubule polymerisation, while CRMP2 phosphorylation loses its affinity for cytoskeletal proteins, leading to microtubule disorganisation inhibition of axonal growth (Nagai *et* *al,* 2016). CRMP2 inactivation can be mediated via a RhoA‐ROCK‐dependent pathway downstream of CSPGs, MAG and Nogo (Rozes Salvador *et* *al,* 2016; Curcio & Bradke, 2018). Genetically preventing phosphorylation of CRMP2 promotes axonal regeneration attributed to suppressing microtubule depolymerisation after optic nerve injury (Kondo *et* *al,* 2019). Pharmacologically preventing phosphorylation of CRMP2 could be another therapeutic avenue.

Less is known about the role and manipulation of the actin cytoskeleton in axon regeneration. Recently however, the ADF/cofilin family of actin‐regulatory proteins that govern actin retrograde flow and dynamic were shown to promote neurite formation (Tedeschi *et* *al,* 2019). Specifically, enhanced actin turnover by ADF/cofilin is critical for axon regeneration in the adult CNS. Thus, actin turnover is a key target for future therapeutic interventions.

We are missing key pieces of the experimental puzzle such as what triggers the switch between growth competent neurons to growth‐restricted mature neurons. Knowing such mechanisms could open a door to new therapeutic interventions. Whole transcriptome sequencing and bioinformatics analysis identified the calcium voltage‐gated channel auxiliary subunit α2δ2 (Cacna2d2) as a one such developmental switch (Tedeschi *et al,* 2016). Cacna2d2 promotes synapse formation and limits axon growth and regeneration in adult mouse DRG neurons (Tedeschi *et* *al,* 2016). Systemic administration of pregabalin to antagonise Cacna2d2 in adult mice after dorsal column lesion resulted in increased axon regeneration by preventing calcium influxes through Cav2 channels (Tedeschi *et al,* 2016). Further, gabapentinoid administration within a month after human SCI improves motor recovery (Warner *et* *al,* 2017), while other anti‐convulsant drugs appear not to show such effects (Warner *et* *al,* 2019). Many different interventions are available that influence the intrinsic growth response of axons which seem to represent only a piece of the puzzle. We are yet to discover how to revert neurons to their embryonic growth state.

### Resupply: trophic support

Neurotrophins facilitate neuron survival, development and function. They may be administered to the damaged spinal cord by direct infusion, biomaterials and *ex vivo* gene therapy (cell transplantation) or by viral vector expression. Brain‐derived growth factor (BDNF), neurotrophin‐3, (NT‐3), nerve growth factor (NGF), fibroblast growth factor (FGF) and glial cell‐derived growth factor (GDNF), for example, are some of the trophic factors that have been investigated. Different neurotrophic factors have differing effects. For example, NT‐3 elicits growth of CST axons, and NT‐3 and NGF both promote extensive DRG‐origin sensory or axon from the reticular formation and red nucleus (Houweling *et* *al,* 1998; Jakeman *et* *al,* 1998; McTigue *et* *al,* 1998; Oudega & Hagg, 1999; Namiki *et* *al,* 2000; Tuszynski *et* *al,* 2002; Blesch & Tuszynski, 2003; Shumsky *et* *al,* 2003; Zhou & Shine, 2003; Oudega *et* *al,* 2019). BDNF, by contrast, has no such effect but rather stimulates sprouting and growth of rubrospinal, reticulospinal, vestibulospinal, raphespinal and motor axons (Nakahara *et* *al,* 1996; Tuszynski *et* *al,* 1996; Grill *et* *al,* 1997; Bradbury *et* *al,* 1998; Bregman *et* *al,* 2002). There are, however, several issues pertaining to continuous infusions of trophic factors, including damage to the tissue at the infusion site, low stability, limited diffusion and inability to cross the blood–spinal cord barrier. Furthermore, the complexity of the spinal networks conveys another limitation of the use of neurotrophic factors. For example, overexpression of NGF in the dorsal horn resulted in overshooting of targets and formed inappropriate connections, resulting in severe hyperalgesia in rats (Tang *et* *al,* 2004). Similar side effects or lack of efficacy have been observed after trophic delivery in human clinical trials of diabetic neuropathy (Apfel, 2002).

Future preclinical investigations should include non‐human primates that include multiple motor and sensory parameters to show whether neurotrophins safe and effective for SCI treatment. It would be invaluable to discover the required combination and temporal administration of growth factors to elicit regrowth of axons through lesions without side effects. Recently, the essential factors required to propel propriospinal axon regeneration across a complete SCI in the adult mouse were discovered (Anderson *et al,* 2018). The growth capacity of mature descending propriospinal neurons was activated with viral vector‐mediated expression of osteopontin, insulin‐like growth factor 1 (IGF1) and ciliary‐derived neurotrophic factor (CDNF) before a SCI. A growth‐permissive substratum was induced with fibroblast growth factor 2 (FGF2) and epidermal growth factor (EGF), and propriospinal axons were chemoattracted with glial‐derived neurotrophic factor (GDNF) delivery via spatially and temporally controlled release from biomaterial depots placed sequentially after SCI (Anderson *et* *al,* 2018). These three mechanisms in combination stimulated propriospinal axon regrowth through astrocyte scar borders and across lesion cores of non‐neural tissue that was associated with increased electrophysiological recordings in tissue below the lesion but did not result in functional improvements. It may be possible that functional recovery in these animals may only be evident if combined with rehabilitation to elicit use‐dependent plasticity.

### Remyelination

The validity of targeting remyelination after SCI is contentious. The myelin sheaths of myelinated axons increase the speed of transmission of action potentials. The importance of this property is overtly evident in multiple sclerosis; however, researchers have recently questioned whether the contribution of oligodendrocyte support and remyelination in pathophysiology is relevant to functional recovery after SCI (Duncan *et* *al,* 2019). After SCI, there is widespread acute oligodendrocyte death and demyelination of axons and it is predicted that remyelination could be important for protecting axons from further degeneration and enhancing conduction (Norenberg *et al,* 2004; Nave, 2010a, 2010b; Almad *et* *al,* 2011). Two approaches are available: transplantation of cells that may directly differentiate into oligodendrocytes and to promote the recruitment and differentiation of endogenous OPCs. Several studies from various laboratories involving transplantation of Schwann cells, OECs and NPCs have demonstrated functional recovery correlates to their ability to promote remyelination (Tetzlaff *et* *al,* 2011; Karimi‐Abdolrezaee & Eftekharpour, 2012). Within these experiments, the contribution of remyelination towards functional recovery is unclear as transplanted cells have many potential mechanisms of efficacy (see 3.2. Replacement: Cellular transplantation). Failure of recruitment of OPCs to the SCI lesion site is associated with the inability of axon remyelination and sensitivity to degeneration (Irvine & Blakemore, 2008). Promoting the recruitment and differentiation of OPCs to the lesion site can is a lesser‐explored avenue (Duncan *et al,* 2019). Various growth factors and hormones can stimulate the differentiation of OPCs *in vitro* but their *in vivo* efficacy is unclear (Plemel *et* *al,* 2014). More recently however, spontaneous locomotor recovery of stepping following contusive SCI was shown to not require oligodendrocyte remyelination despite the high level of endogenous remyelination that occurs after SCI (Duncan *et* *al,* 2018). This finding raises doubts whether therapeutic targeting of oligodendrocyte remyelination is a worthwhile target for clinical translation, a debate we cannot yet conclude at this time. The contribution of Schwann cell myelination may possibly be a more meaningful target. Neuregulin‐1 was found to drive trans‐differentiated of OPCs into Schwann cells, and these cells were essential for Schwann cell‐mediated remyelination following SCI (Bartus *et* *al,* 2016). Furthermore, preventing neuregulin‐1/ErbB signalling‐controlled transformation of CNS progenitors into myelinating Schwann cells resulted in negative consequences for functional recovery following spinal cord contusion in mice (Bartus *et* *al,* 2019). Another consideration is interspecies differences. The majority of intact axons in chronically injured rats exhibit remyelination, albeit with some structural abnormalities (Powers *et* *al,* 2012). Neither was chronic demyelination observed in spared axons after SCI in mice (Lasiene *et* *al,* 2008). It is unclear whether this is also the case in humans and primates or whether remyelination contributes to the same functions as it does in rodents, owing to the need for more investigations on this topic.

### Rehabilitation: neurorehabilitation, neuronal activity and electrical stimulation

Recent evidence has documented that, in the right context, rehabilitation can play a critical role in regeneration and plasticity (Loy & Bareyre, 2019). Various forms of rehabilitation can enhance the recovery of motor functions after SCI in mice, rats and cats. Rehabilitation is important to prune and refine circuits that are relevant to the motor task (Ichiyama *et* *al,* 2008). Moreover, rehabilitation is also reported to stimulate the local production of neurotrophins (Ying *et* *al,* 2005; Cote *et* *al,* 2011b), modulate multiple neurotransmitter systems (Edgerton *et* *al,* 2004) and enhance sprouting of compensatory relay networks (Courtine *et* *al,* 2009; van den Brand *et* *al,* 2012). Animal models of rehabilitation can come in multiple forms of forced or voluntary physical exercise and environmental enrichment as well as on a spectrum of general to more specific approaches. Increasing general activity of rodents through environmental and social enrichment such as wheel running, multiple housing and the addition of climbing spaces, improved bedding and crawl toys all significantly increase BBB locomotor scores (Berrocal *et* *al,* 2007; Loy *et* *al,* 2018). Surprisingly, environmental enrichment prior to a SCI in mice and rats mimicked the degree of proprioceptive afferent neurons regeneration and functional recovery to a conditioning lesion (Hutson *et* *al,* 2019). The regenerative response correlated with Creb‐binding protein (Cbp)‐mediated histone acetylation and epigenetic changes inducing signalling pathways involved in neuronal activity, calcium modulation and the regenerative programme in proprioceptive neurons (Hutson *et al,* 2019). A comparative analysis of rats with incomplete SCI subjected to three forced‐exercise rehabilitation paradigms (quadrupedal treadmill training, swim training and stand training) differentially improved recovery after spinal cord injury (Hutchinson *et* *al,* 2004). Quadrupedal treadmill‐trained rats showed reduced allodynia and restored sensation; swim training reduced allodynia and stand training had no benefit. This indicates that stand training may not be intensive enough above spontaneous movement. Similarly, quadrupedal treadmill training was superior to hindlimb step training in terms of electrophysiological and kinematic limb analysis and the number of propriospinal labelled neurons above and below the lesion (Shah *et* *al,* 2013). Utilising task‐specific rehabilitative strategies such as skilled paw retrieval, horizontal ladder training or hindlimb‐specific training makes intuitive sense in that the best way to relearn a given task is to specifically train for that task. For example, hindlimb‐specific treadmill training can be used after thoracic contusion to significantly improve that lost function although this negatively influenced forelimb–hindlimb inter‐coordination (Griffin *et* *al,* 2020). It is apparent that emphasising a particular behaviour can negatively affect the performance of another, which suggests that reassignment of CNS resources is limited (Fawcett & Curt, 2009). For example, rats trained at skilled paw reaching improved in that task but this worsened their performance at walking on ladders, and general environmental enrichment rehabilitation can improve locomotor function, but extinguish skilled tasks (Girgis *et* *al,* 2007; Garcia‐Alias *et* *al,* 2009). Likewise, spinally transected cats could be trained in weight support or step; however, training one behaviour would extinguish the other (De Leon *et* *al,* 1998a, 1998b). We are still rudimental in our understanding of how rehabilitation influences regeneration, neuroplasticity and motor recovery following SCI. Several studies reported exercise‐induced plasticity of various spinal tracts, particularly the serotonergic and reticulospinal tracts (Engesser‐Cesar *et* *al,* 2007; Asboth *et* *al,* 2018; Loy *et al,* 2018). Conversely, an equalling number reported no such ability and proposes the role of rehabilitation in shaping newly formed connections by spontaneous mechanisms or plasticity‐promoting treatments (Garcia‐Alias *et al,* 2009; Maier *et* *al,* 2009; Wang *et* *al,* 2011a; Alluin *et* *al,* 2014). Regardless of these contrasting reports, it is clear that in general, a high‐intensity programme and is key to providing robust motor function improvements in rats (Loy & Bareyre, 2019).

It should be briefly mentioned that very limited recovery after anatomically complete injuries with rehabilitation is observed (Ilha *et* *al,* 2011). However, since the spinal circuitry below the lesion remains active, it can be activated through pharmacological and electrical modulation of the central pattern generator and lead to weight‐bearing stepping in rats with completely transected spinal cords (Courtine *et al,* 2009).

We are also rudimental in our understanding of how electrical stimulation and neuronal activity affect regenerative processes and investigations are ongoing. Brief electrical stimulation accelerates axon outgrowth in models of peripheral nerve injury (Gordon, 2016), and spinal cord stimulation in human SCI patients has shown efficacy in randomised clinical trials over several decades (Sdrulla *et* *al,* 2018). Exposure to an electrical field *in vitro* increased neurite outgrowth of embryonic and adult neurons, and electrical stimulation of the sciatic nerve *in vivo* increased central DRG axon regeneration comparable to a conditioning lesion (Wood & Willits, 2006; Udina *et* *al,* 2008). Conversely, electrical stimulation has also been associated with axon growth inhibition. Electrical activity strongly inhibited axon outgrowth in cultured adult DRG neurons, an effect depended on the L‐type voltage‐gated Ca^2+^ channel current and involved transcriptional changes (Enes *et* *al,* 2010). Knockout of the L‐type voltage‐gated Ca^2+^ channel current in adult mice was sufficient to boost the growth ability of DRG neurons *in vivo* after central lesioning (Enes *et al,* 2010). Likewise, suppressing synaptic function through gabapentinoid‐mediated blockade of the voltage‐gated channel auxiliary subunit α2δ2 also induces axon regeneration the adult CNS (Tedeschi *et al,* 2016). This counter‐position hypothesises that electrical stimulation suppresses growth by triggering an increase in intracellular calcium, and conversely, a lack of electrical activity within axotomised neurons may recapitulate development and promote axon regeneration (Hilton & Bradke, 2017). It is possible that temporal regulation and the effects of conditioning neurons may account for the contrary evidences and is therefore an exciting prospect for further investigations.

## Combinatory approaches

**Figure 2:**
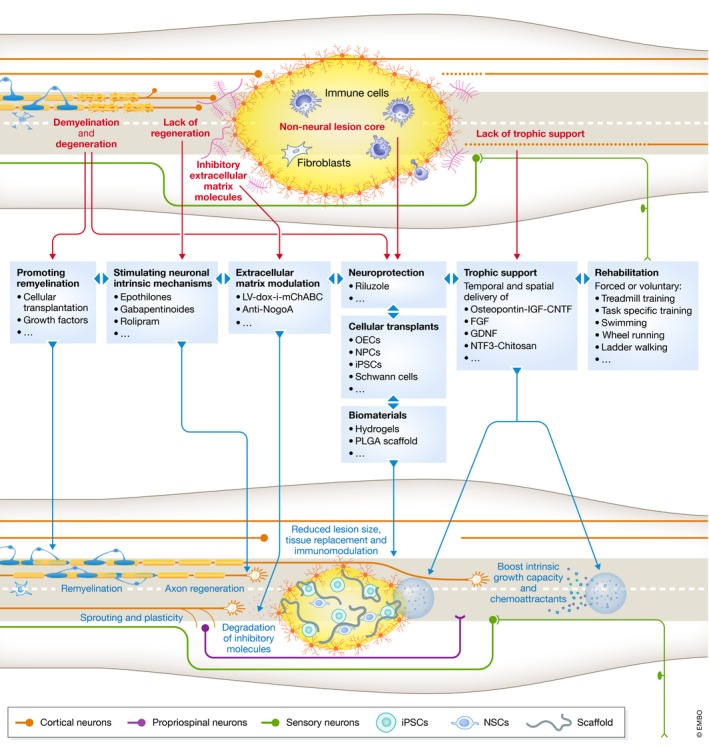
Combinatory therapies for spinal cord injury Experimental spinal cord injury research has resulted in a multitude of individual therapeutic possibilities yet recovery after injury remains incomplete. Combinatory approaches to address the seven targets will need to be a focus of translation studies. Tissue and cellular transplants will replace lost cells, among other regenerative functions. Removal of inhibitory factors such as CSPGs allows for enhanced axonal growth. Targeting neuron‐intrinsic mechanisms enhance intrinsic regenerative response which can then be directed through resupply of trophic support. Remyelination of demyelinated axons may improve axonal conduction and survival. Finally, rehabilitation functions in circuit remodelling and strengthens beneficial connections.

While individual therapies have shown promise in preclinical models, recovery remains incomplete and it is becoming apparent that focusing a singular barrier to repair is not going to facilitate successful and functional recovery of SCI in humans. Conversely, it will require the combination of various treatment strategies that address diverse aspects of SCI pathology. Removal of growth inhibitory molecules as well as boosting the intrinsic growth response will be required. Tissue or cell transplants may be required to replace tissue, act as a permissive bridge and/or facilitate repair. Supplying trophic support will improve survival of cells and direct axonal growth. Lastly, neurorehabilitation will function to augment the functional recovery of remodelling circuits (Tables 1, 2, 3, 4, 5). This section will summarise currently published studies that have combined therapies in SCI models (Fig 2).

**Table 1:** Combinations of cell transplants with neurotrophins in preclinical experiments

Transplant type	Growth factor	Injury model	Outcome	Reference
Schwann cells in semipermeable guidance channels	BDNF and NT‐3 delivery via minipump	Female Fischer rats 160‐190 g Mid‐thoracic transection Acute treatment	BDNF and NT‐3 infusion enhanced propriospinal axonal regeneration and, more significantly, promoted axonal regeneration of specific distant populations of brain stem neurons into grafts at the mid‐thoracic level in adult rat spinal cord	Xu *et al* (1995a)
Rat intercostal nerve	aFGF in fibrin glue	Adult Sprague Dawley rats T8 transection Acute treatment	Hindlimb function improved progressively during the first 6 months, as assessed by two scoring systems. The corticospinal tract regenerated through the grafted area to the lumbar enlargement, as did several bulbospinal pathways	Cheng *et al* (1996)
Rat fibroblasts	Fibroblasts genetically modified to secrete NGF, BDNF, NT‐3 or bFGF	Adult rats Acute SCI	Sensory neurites of dorsal root origin extensively penetrated NGF‐, NT‐3‐ and bFGF‐producing grafts, whereas BDNF‐secreting grafts elicited no growth responses. Putative noradrenergic neurites also penetrated NGF‐secreting cell grafts. Local motor and corticospinal motor axons did not penetrate any of the neurotrophic factor‐secreting grafts	Nakahara *et al* (1996)
Rat PNG	IGF, bFGF or TGFβ in gelfoam	Adult rats, C3 hemisection Chronic treatment (1 month)	Greatest increase of axonal regeneration by TGFβ	Houle *et* *al* (1996)
Rat PNG	NT‐3, BDNF or CNTF in gelfoam	Adult female Sprague Dawley rats (200‐225 g) C2/3 dorsal hemisection Treatment 4 weeks after injury	Growth factors were required to promote axonal growth into the PN graft. CNTF most effective. Functional testing not done	Ye and Houle (1997)
Foetal spinal cord tissue	Gel foam soaked with NT‐3 and BDNF in gelfoam	Adult male and female Sprague Dawley rats (200–250 g) T6 hemisection Acute treatment	Application of either transplants or neurotrophic factors partially reverses the axotomy‐induced atrophy in rubrospinal neurons, but that both interventions together reverse the atrophy completely.	Bregman *et* *al* (1998)
Minced rat PNG	BDNF, NT‐3 and GDNF collagen matrix in cavity	Adult female rats 150–200 g T10 dorsal hemisection Chronic treatment	Combination therapy led to sustained regeneration of the CST	Ferguson *et* *al* (2001)
Autologous PNG	Gelfoam soaked with GDNF	Adult female Sprague Dawley rats 225–250 g C3 hemisection Acute treatment	Sevenfold increase in the number of regenerating neurons after GDNF‐treatment.	Dolbeare and Houle (2003)
Fibroblasts	Fibroblasts genetically modified to secrete NT‐4/5	Adult Fisher 344 rats Dorsal hemisection or complete transections at the mid‐thoracic level	Motor axons, coerulospinal, reticulospinal and propriospinal axons responded to NT‐4/5 delivery after thoracic spinal cord injury with significantly increased axonal penetration into NT‐4/5 secreting grafts compared to control grafts. Axonal growth beyond NT‐4/5‐producing grafts and functional recovery were not observed	Blesch *et al* (2004)
Fibroblasts	Fibroblasts genetically modified to secrete BDNF and NT‐3	Adult female Sprague Dawley rats 225–250 g T8/9 moderate contusion Acute treatment	BDNF/NT‐3 rats recovered from areflexic bladder earlier showed decreased micturition pressure and fewer episodes of detrusor hyperreflexia, as well as improvements to hindlimb function compared to untreated	Mitsui *et al* (2005)
Human umbilical cord blood cells	BDNF	Adult male Sprague Dawley Rats 300‐350 g T9 contusion NYU weight‐drop device 10 g weight	8 weeks after transplantation, the HUCBs with BDNF transplanted group had improved BBB scores, than the other groups	Kuh *et* *al* (2005)
Bone marrow stromal cells	Lentiviral NT3 expression caudal	Adult Female Fischer 344 rats Dorsal column lesion C2/C3 Acute treatment	LV‐NT‐3 allowed regenerating axons to grow beyond a PNG	Taylor *et al* (2006)
Mouse embryonic stem cell‐derived NPCs	NT‐3 + PDGF in fibrin/heparin scaffolds	Adult female Long–Evans rats 250–275 g T9 dorsal hemisection Subacute treatment (2 weeks)	Combination enhanced transplanted cell survival and increased the number of NPC‐derived NeuN‐positive neurons 8 weeks after transplantation. All experimental groups treated with NPCs exhibited an increase in behavioural function 4 weeks after transplantation. In a subset of animals, the cells formed tumours	Johnson *et al* (2010a)
Mouse embryonic stem cell‐derived NPCs	NT‐3 + PDGF in fibrin/heparin scaffolds	Adult Long–Evans female rats 250–275 g Dorsal hemisection T9 Subacute model (2 weeks)	The combination enhanced the total number of ESNPCs present in the spinal cord lesion 2 weeks after injury. No functional scoring reported.	Johnson *et* *al* (2010b)
Schwann cells and NSCs	NSCs genetically enhanced expression of NT‐3 in gelfoam	Adult female Sprague Dawley rats Complete T10 transection Acute treatment	Significantly improved relay of the cortical motor evoked potential and cortical somatosensory evoked potential as well as ameliorated hindlimb deficits. Neuroprotection and outgrowth of serotonergic firers	Wang *et* *al* (2011b)
E14 rat NSC human ESC‐derived NSC cell lines 566RSC, HNES7 in fibrin matrix	BDNF, NT‐3, PDGF‐AA, IGF‐1, EGF, bFGF, aFGF, GDNF, GDNF, HGF, calpain inhibitor MDL28170	Adult female Fischer 344 rats T3 complete transection 2 mm or C5 hemisection Treatment 2 weeks after injury	Extensive axonal outgrowth rostral and caudal from the transplant. Human and rat cells performed similarly. Combination greatly improved functional recovery	Lu *et* *al* (2012)
Adult rat brain NPCs supplemented with EGF, FGF and heparin	PDGF in hyaluronan‐based hydrogel	Adult female Wistar rats 250–300 g T2 26 g clip compression Acute treatment	The combination of PDGF with cells protected oligodendrocytes around the lesion. The combination reduces number of errors on ladder task	Mothe *et* *al* (2013)
Human IPSCs in fibrin matrix	BDNF, NT‐3, PDGF‐AA, IGF‐1, EGF, bFGF, aFGF, GDNF, GDNF, HGF, calpain inhibitor MDL28170	Adult female athymic nude rats and adult SCID mice C5 lateral hemisection Treatment 2 weeks after injury	Extensive outgrowth from grafted cells, but not functional recovery. Few cells were positive for mature markers	Lu *et al* (2014)
Human iPSC‐derived OPCs	PDGF in hyaluronan/methylcellulose hydrogel	Adult female Sprague Dawley rats 300 g T2 26 g clip compression Acute treatment	The combination promoted cell survival and differentiation of the cells. No functional recovery observed	Fuhrmann *et* *al* (2016)

**Table 2:** Combinations of cell transplants with anti‐inhibitory therapies in preclinical experiments

Transplant type	Anti‐inhibitory therapy	Injury model	Outcome	Reference
Human Schwann cells	IN‐1 antibody + aFGF–fibrin glue	Adult female athymic nude rats 145–165 g T8 transection Acute treatment	Human SC grafts alone do not support the regeneration of injured CST fibres and do not prevent die‐back. Grafts plus IN‐1 antibody‐containing supernatant support some sprouting but die‐back continues. Grafts plus aFGF–fibrin glue support regeneration of some fibres into the grafts and reduce die‐back	Guest *et al* (1997)
PNG	BDNF or ChABC	Adult female Sprague Dawley rats 200–250 g T11 hemisection Acute treatment	BDNF did not improve axonal regeneration; however, ChABC resulted in significant increase in the number of regenerated Clarke's nucleus neurons	Yick *et al* (2000)
Schwann cell‐seeded channels	ChABC intraparenchymal infusion	Adult female Fischer rats T8 hemisection Acute treatment	Significant anatomical evidence of regeneration through the graft compared with that seen without ChABC treatment	Chau *et al* (2004)
Schwann cell matrigel guidance channels + olfactory ensheathing glia grafts	ChABC osmotic pump delivery	Adult female Fischer 344 rats 165–180 g Complete thoracic T8 transection Acute treatment	Increased 5HT fibres exiting bridge caudally. Functional recovery which was absent without ChABC application	Fouad *et al* (2005)
NSCs	ChABC pretreatment	Adult female Sprague Dawley rats 230–250 g T10 contusion (10 g weight drop) Acute treatment	Combined treatment significantly induced the outgrowth of a greater number of growth‐associated protein‐43‐positive fibres at the lesion epicentre, compared with NSPC transplantation alone	Ikegami *et* *al* (2005)
PNG	ChABC intrathecal infusion at lesion site	Adult female Sprague Dawley rats 225–250 g C5 dorsal quadrant aspiration Acute treatment	Combination promotes significant axonal regeneration beyond the distal end of a PN bridge back into the spinal cord and that regenerating axons can mediate the return of useful function of the affected limb	Houle *et al* (2006)
Schwann cell‐filled guidance channels + OEC implant	ChABC intraspinal injections	Adult female Fischer 344 rats Complete transection Acute treatment	Regeneration of many fibre tracts and the combination was associated with significantly improved locomotor recovery	Vavrek *et al* (2007)
PNG	Intraspinal ChABC microinjection	Adult female Sprague Dawley rats 225–250 g C5 dorsal quadrant aspiration Acute treatment	More regenerating axons to exit a PNG and reenter spinal cord tissue than saline injections	Tom and Houle (2008)
PNG	mRNA‐mediated knockdown of XT‐1	Adult female Sprague Dawley rats (200–225 g). Thoracic dorsal transection	1.4‐fold reduction in GAG‐side chains of chondroitin sulphate or heparin sulphates‐PGs. Ninefold increase in length and a fourfold increase in density of ascending axons growing through the nerve graft and scar tissue present at the rostral spinal cord	Hurtado *et al* (2008)
NPCs	ChABC + EGF, bFGF and PDGF‐AA	Adult female Wistar rats 250 g T7 Clip compression Chronic treatment	Combined strategy promoted the axonal integrity and plasticity of the corticospinal tract and enhanced the plasticity of descending serotonergic pathways and significantly improved neurobehavioural recovery	Karimi‐Abdolrezaee *et al* (2010)
PNG	ChABC injections at day 1 and 1 week later	Adult female Sprague Dawley rats 240–300 g C2 lateral hemisection Acute treatment	Combination with a peripheral nerve autograft, ChABC treatment resulted in lengthy regeneration of serotonin‐containing axons and other bulbospinal fibres and remarkable recovery of diaphragmatic function compared to alone	Alilain *et al* (2011)
Adult rat NPCs	Nogo‐66 receptor protein (blocker) + bFGF, EGF, PDGF via osmotic pump	Adult female Sprague Dawley 200–300 g T8 complete transection Acute treatment	Transplanted cells survive longer with the growth factors; NgR had no effect on their survival. NgR increases myelination. Combination did not improve sprouting or functional recovery	Guo *et al* (2012)
PNG + fibrin glue + aFGF	ChABC microinjection rostral and caudal	Adult male Sprague Dawley rats 225–250 g T8 transection Acute treatment	Remarkably lengthy regeneration of certain subtypes of brainstem and propriospinal axons across the injury site. Restoration of supraspinal bladder control	Lee *et al* (2013)
PNG	ChABC + acidified FGF intraspinal injection mixture	Adult Sprague Dawley rats 225–250 g Severe T8 contusion 250 KDyne Acute and chronic treatment	The combination enhanced integration between host astrocytes and graft Schwann cells, allowing for robust growth. Axons did not enter/exit the graft without ChABC and aFGF. Limited compared to the acute scenario. Combination leads to functional improvements	DePaul *et al* (2017)

**Table 3:** Combinations of anti‐inhibitory therapies with growth factors in preclinical experiments

Anti‐inhibitory therapy	Growth factor	Injury model	Outcome	Reference
Thermostabilised ChABC	NT‐3 lipid microtubes embedded in agarose gel for both molecules	Adult male Sprague Dawley rats T10 dorsal hemisection Acute treatment	Animals treated with ChABC in combination with sustained NT‐3 delivery showed significant improvement in locomotor function and enhanced growth of cholera toxin B subunit‐positive sensory axons and sprouting of serotonergic fibres	Lee *et al* (2010)
ChABC intraspinal injections	NT‐3 and NR2D expression	Adult female Sprague Dawley rats 200 g T8 Lateral Hemisection Acute treatment	Animals receiving combined therapy displayed the most improved body stability and inter‐limb coordination. Only animals with the full combination treatment recovered consistent multisynaptic responses in these motor neurons indicating formation of a detour pathway around the injury site	Garcia‐Alias *et al* (2011)
Nogo‐66 receptor protein (blocker)	bFGF, EGF, PDGF via osmotic pump +adult rat NSCPs	Adult female Sprague Dawley 200–300 g T8 complete transection Acute treatment	Transplanted cells survive longer with the growth factors; NgR had no effect on their survival. NgR increases myelination. Combination did not improve sprouting or functional recovery	Guo *et al* (2012)
ChABC	NGF in electrospun scaffold	Adult female Sprague Dawley rats T9/T10 complete transection Acute treatment	Improved BBB scores compared to implant only	Colello *et al* (2016)
NEP1‐40 (Nogo antagonist), ephrin‐B3 and Sema4D receptor	Collagen‐binding BDNF and NT‐3 cAMP in functionalised collagen scaffold	Adult female Sprague Dawley rats 200–230 g T10 complete transection Acute treatment	Full combinatorial therapy exhibited the greatest advantage in reducing the volume of cavitation, facilitating axonal regeneration and promoting neuronal generation. Neurons generated in the lesion area could form the neuronal relay and enhance the locomotion recovery	Li *et al* (2016)
Anti‐Nogo‐A	NT‐3 delivery by nanoparticles	Adult female Sprague Dawley rats T1/2 clip compression Acute treatment	Increased anatomical improvements in both treatments individually. Only functional improvements in the combination group	Elliott Donaghue *et al* (2016)

**Table 4:** Combination treatments involving targeting the intrinsic growth response in preclinical experiments

Therapy	Combination	Injury model	Outcome	Reference
Rolipram, cAMP	Rat Schwann cell transplantation	Adult female Fischer 344 rats 160–180 g T8 moderate contusion injury Acute treatment	The combination of rolipram and cAMP had the greatest effect on cAMP levels, axonal sparing, myelination and locomotor function	Pearse *et al* (2004b)
Rolipram (minipump drug delivery)	Rat Schwann cells expressing D15A (BDNF + NT3)	Adult female Fischer 344 rats 180–200 g T8 moderate contusion injury MASCIS weight drop Subacute treatment (2 weeks)	Compared to the single treatments, the combination led to the largest SC grafts, the highest numbers of serotonergic fibres in the grafts, and increased numbers of axons from the reticular formation below the lesion/implant area and provided the greatest improvement in hindlimb function	Flora *et al* (2013)
Scar‐targeted liposomes containing docetaxel	BDNF, aFGF	Adult female Sprague Dawley 220–230 g T10 contusion 50 mm Height MASCIS impactor Acute treatment	The combined application of GFs and DTX supported neuroregeneration by improving neuronal survival and plasticity, rendering a more permissive extracellular matrix environment with improved regeneration potential. In addition, our combination therapy promoted axonal regeneration via moderation of microtubule function and mitochondrial transport along the regenerating axon. Significantly improved BBB score	Wang *et al* (2018)
Taxol and cetuximab in collagen scaffold	Cetuximab (EGFR signalling antagonist)	Adult female Sprague Dawley rats 190–210 g Complete T10 transection Acute treatment	Combined functional scaffold implantation significantly increased neural regeneration to reconnect the neural network and improved functional recovery	Fan *et al* (2018)

**Table 5:** Combinations of therapies with rehabilitation in preclinical experiments

Combination	Form of rehabilitation	Injury model	Outcome	Reference
Autologous bone marrow stem cells	Swim training 60 min a day 6 days/week	Adult male Wistar rats 350 g NYU impactor contusion: 10 g 25 mm height Cell transplant 48 h after injury	The combination of bone marrow stem cell therapy (CD45 (+)/CD34 (−)) and exercise training resulted in significant functional improvement in acute spinal cord injury	Carvalho *et al* (2008)
ChABC infusion	Forepaw reaching and grasping rehabilitation	Adult male Lister Hooded rats 250–300 g C4 dorsal funiculus cut Acute ChABC treatment	Synergistic effect compared to either intervention alone	Garcia‐Alias *et al* (2009)
Anti‐Nogo‐A antibody	Bipedal and quadrupedal treadmill training—1 week after injury	Adult female Sprague Dawley rats 200–250 g T‐Lesion T8 Acute treatment	Lack of synergistic effect with the combination	Maier *et al* (2009)
Single lumbar ChABC injection	Voluntary wheel running	Adult female C57BL/6 mice Moderate contusion Ohio State ESCID impactor ChABC treatment 1 week after injury	Rehabilitation did not improve functional recovery	Jakeman *et* *al* (2011)
5 ChABC intraspinal injections over 10 days beginning 1 month after injury	Task‐specific paw reaching beginning 1 month after injury	Adult male Lister Hooded rats 150–200 g C4 dorsal hemisection Chronic treatment	Significant improvement to paw reaching task with combination but only after ChABC infusion	Wang *et al* (2011a)
Anti‐Nogo‐A infusion into intrathecal space for 2 weeks followed by 5 intraspinal ChABC injections over 10 days	Multitask rehabilitation: Seed‐reaching task and ladder walking beginning 4 weeks after the lesion	Adult male Lister Hooded rats 150–200 g C4 dorsal hemisection	Both single treatments produced increases in sprouting and axon regeneration, but the combination treatment produced greater increases	Zhao *et al* (2013)
ChABC, PDGF, bFGF, EGF subarachanoid infusion for 7 days	Daily quadrupedal treadmill training 15 min a day for 3 weeks	Adult female Wistar rats 250–275 g Rat T7 23.8 g clip compression 1 min. Acute treatment 4 days after injury	Combined therapy significantly enhanced the neuroanatomical plasticity of major descending spinal tracts such as corticospinal and serotonergic‐spinal pathways. Structural changes did not translate to an additional long‐term improvement of locomotor parameters	Alluin *et al* (2014)
AAV10‐NT‐3 intraspinal injections	Spinal electromagnetic stimulation every 2 days (2.8 T, 0.2 Hz, 35 min) Swimming and exercise ball training	Adult female Sprague Dawley rats 210 g Rat T10 contusion 150 KDyne IH impactor Acute and chronic treatments; sequential electrical stimulation	Acutely, the combination significantly improves electrophysiology recordings, narrow beam task, error ladder task and Catwalk gait parameters Chronic treatment also improved electrophysiology recordings when all treatments are combined.	Petrosyan *et al* (2015)
ChABC intrathecal infusion 6 weeks after injury for 7 days	Quadrupedal treadmill exercise weeks 6–14; 30 min a day, 5 days a week	Adult female Sprague Dawley rats 200–220 g Severe 250 KDyne contusion IH impactor Acute treatment	Increases in spared tissue and neuronal fibre regeneration. No associated improvement to motor functions.	Shinozaki *et al* (2016)
Anti‐Nogo‐A 2‐week continuous infusion	Sequential (3 weeks after injury/1 week after last treatment) hindlimb bipedal treadmill training for 8 weeks, 5 days a week	Adult female Sprague Dawley rats 200–250 g T‐Shaped lesion Transection of dorsomedial, dorsolateral, ventromedial CST	Sequential training showed superior recovery of motor function. No improvement when treated in parallel	Chen *et al* (2017)

### Combining cellular transplants with neurotrophins

Early attempts at transplantation of cells into the damaged spinal cord promoted some degree of axonal regeneration into the graft. However, axons fail to grow beyond the graft, transplants typically lack appropriate orientation, and the hostile adult spinal cord environment limits transplant survival and/or differentiation. The logical addition of neurotrophins was one of the first combinatory strategies to address these problems and many studies have since been conducted over the last three decades (Table 1). The first attempt at this strategy utilised Schwann cells in semipermeable guidance channels in combination with BDNF and NT‐3 delivery via a minipump in a thoracic transection model (Xu *et* *al,* 1995a). BDNF and NT‐3 infusion enhanced propriospinal axonal regeneration and, more significantly, promoted axonal regeneration of specific distant populations of brain stem neurons into grafts at the mid‐thoracic level in adult rat spinal cord. In the following years, many studies reported that combining growth factor delivery with a PNG is capable of allowing axons to extend beyond the graft, although this was often not associated with improvement to behavioural measures (Cheng *et* *al,* 1996, 2004; Ye & Houle, 1997; Dolbeare & Houle, 2003). This may reflect a lack of beneficial integration of the cells and a limitation of PNGs over other transplant types. Many combinatory strategies for PNGs have utilised FGF (Table 1). It appears that FGF is an important growth factor to include with cellular transplantation. Firstly, FGF tends to stimulate axonal elongation in a rectilinear rather than a branching pattern, which is critical for long‐distance regrowth. Secondly, FGF tends to act on reactive astrocytes to cause them to change their shapes into a more primitive bipolar morphology, which is critical to allow axons to pass the graft–host interface (Zhou *et* *al,* 2018).

It is apparent that the transplant and growth factor delivery methods are important considerations and they need to be spatially and temporally organised. Cell and drug delivery by injection or loaded gelfoam are substandard due to washing away of cells and short‐lived release of the factors, respectively. Osmotic pumps ensure long‐lasting local delivery but are invasive and can cause local tissue damage. *Ex vivo* gene therapy delivery of cells to secrete growth factors is limited for the recurring reason that the high concentration gradient of growth factors at the transplant site does not allow axons to leave the graft (Table 1; Nakahara *et al,* 1996; Blesch *et* *al,* 2004; Mitsui *et* *al,* 2005). This may possibly be overcome by further expression of growth factors beyond the graft such as what was observed after lentiviral NT‐3 administration beyond a bone marrow stromal cell transplant (Taylor *et* *al,* 2006). Non‐regulated viral vector gene expression of growth factors can allow for spatial expression of specific growth factors but is limited in that continued delivery after resolution is likely to be disadvantageous. The development of various natural and synthetic polymer biomaterials is advancing as a promising way to circumvent the limitations of the other delivery methods although they each also have benefits and limitations (Fuhrmann *et* *al,* 2017).

Fibrin matrix‐delivery of two human ESC‐derived NPCs with a cocktail of ten growth factors after severe SCI allowed for extensive axonal outgrowth of the grafted cells rostral and caudal from the transplant and the combination improved functional recovery (Lu *et al,* 2012). This experiment was later repeated using human IPSCs. A similar level of extensive axonal outgrowth from the grafted cells was present, although in this case there was no improvement to motor functions which may imply an inability of IPSCs to integrate into the hose tissue to form functional connections (Table 1; Lu *et* *al,* 2014). Extensive axon growth is not always beneficial as axons may overshoot and oversupply targets, leading to spasticity, neuropathic pain and worsened motor outcome. Another issue of the combination of transplants with growth factors is that growth factors may enhance uncontrolled differentiation of NSCs into astrocytes which may lead to allodynia or pluripotent cells to overproliferate or form tumours (Hofstetter *et* *al,* 2005). For example, in a subset of animals, the combination of ESC‐derived NPCs combined with platelet‐derived growth factor (PDGF) and NT‐3 led to tumour formation by 8 weeks after transplantation (Johnson *et* *al,* 2010a). Therefore, terminally differentiated cells such as Schwann cells or OECs may be a more suitable option. There is currently no gold standard cell type or growth factors, and combinations of the two for clinical translation and so it may be short‐sighted to progress with clinical trials before this information is attained.

### Combination of cellular transplants with anti‐inhibitory therapies

Removing inhibitory elements through various means in conjunction with cellular transplantation has been investigated to enable regeneration in a more permissive environment (Table 2). Through such interventions, regenerating axons are capable of exiting the graft and are likely to form functional synaptic contacts. The first of these studies was in the form of administration of the IN‐1 antibody (anti‐Nogo‐A antibody) with human Schwann cells with aFGF–fibrin glue after acute thoracic transection in rats. Inclusion of the antibody and aFGF were necessary to support sprouting and reduce axon die‐back (Guest *et* *al,* 1997). Studies utilising ChABC later became a major focus in subsequent attempts that led to axonal growth beyond the grafts of Schwann cells, PNGs, OECs and NPCs (Chau *et* *al,* 2004; Houle *et* *al,* 2006; Vavrek *et* *al,* 2007; Tom & Houle, 2008; Alilain *et* *al,* 2011; Lee *et* *al,* 2013; DePaul *et* *al,* 2017). Unlike the combination of PNGs with neurotrophins, combinations with PNGs with ChABC appear to have much more pronounced effects on regeneration and functional recovery, indicating that unmasking neuroplasticity is crucial for the formation of functional connections (Yick *et* *al,* 2000; Houle *et al,* 2006; Alilain *et al,* 2011; Lee *et al,* 2013; DePaul *et al,* 2017). Likewise, similar subjective conclusions could be drawn for Schwann cells, OECs or NPCs, although direct comparisons to these relative roles have not been drawn (Table 2; Chau *et al,* 2004; Fouad *et* *al,* 2005; Vavrek *et al,* 2007; Karimi‐Abdolrezaee *et* *al,* 2010). Rather, growth factors do play an important role in the survival of the graft. Co‐treatment of Nogo receptor blocking peptide (NgR) with delivery of bFGF, EGF and PDGF with adult NSCPs could overcome substantial cell loss; the growth factors increased the survival of the transplanted cells while NgR had no effect on their survival, and NgR increased axonal regeneration and myelination (Guo *et al,* 2012). In another study, only the combination of NPCs with ChABC and the growth factors EGF, bFGF and PDGF‐AA could significantly improve neurobehavioural recovery without enhancing aberrant synaptic connectivity (Table 2; Karimi‐Abdolrezaee *et al,* 2010). Yet, all of these results were achieved from infusions or microinjections of ChABC enzyme which has suboptimal stability and activity *in vivo* compared to gene therapy approaches (Lee *et* *al,* 2010). The combination of the dox‐inducible LV‐mChABC vector with other therapies is yet to be reported. It would be interesting to combine such tuning of plasticity and delivery of growth factors.

### Combining anti‐inhibitory therapies with neurotrophins

Combing neurotrophins with anti‐inhibitory therapies was conceived to promote axonal growth in a newly permissive environment (Table 3). The first attempt used ChABC with NT‐3‐loaded lipid microtubes embedded in agarose gel following T10 dorsal hemisection in rats (Lee *et al,* 2010). Animals treated with ChABC in combination with sustained NT‐3 delivery showed a significant improvement in locomotor function and enhanced growth of cholera toxin B subunit‐positive sensory axons as well as sprouting of serotonergic fibres. This result was replicated by an independent group in which animals receiving combined therapy displayed the most improved body stability and inter‐limb coordination (Garcia‐Alias *et* *al,* 2011). Only animals with the full combination treatment recovered consistent multisynaptic responses in these motor neurons indicating formation of a detour pathway around the injury site.

Similar to ChABC, anti‐Nogo‐A antibody in combination with NT‐3 delivery by nanoparticles in an adult rat clip compression model led to increased anatomical improvements in both treatments individually but only functional improvements in the combination group (Elliott Donaghue *et* *al,* 2016). This combinatory approach may be limited in the context of complete transection injuries since increasing neuronal plasticity through anti‐inhibitory therapies largely relies on spared circuitry and the formation of relay networks (Bradbury & McMahon, 2006). For example, in a model of complete transection, co‐treatment of Nogo receptor blocking peptide (NgR) with delivery of bFGF, EGF and PDGF with adult NSCPs did not have an effect on functional recovery or regeneration (Table 3; Guo *et al,* 2012). The growth factors, not the NgR, improved survival of the transplanted cells, while the NgR enhanced axonal regeneration and myelination. However, this is not always the case. Impressively, the combination of ChABC with neurotrophin delivery has also reported to be therapeutic in complete transection models in which ChABC typically has a very limited therapeutic benefit. ChABC and NGF delivery from an electrospun scaffold following thoracic complete transection in rats displayed a significant improvement in BBB functional recovery and that the electrospun matrix is an important factor in this observation (Colello *et* *al,* 2016). Likewise, antagonism of Nogo, EphrinB3 and sema4D receptor combined with the growth factors BDNF, NT‐3 and cAMP administration after complete transection reduced the volume of cavitation, facilitated axonal regeneration and promoted neuronal generation leading to enhanced locomotion recovery (Table 3; Li *et* *al,* 2016). How does this occur? In the latter study, it was argued that new‐born neurons generated at the lesion area could form the neuronal relay and enhance the locomotion recovery, but this is yet to be confirmed. There clearly are many variables between these experiments that complicate the interpretations of the outcomes, but further investigations and replications should result in more clarification.

### Combination treatments involving targeting the intrinsic growth response

A less explored area of research is enhancing the regenerative programme of injured neurons through targeting intracellular pathways and mechanisms as a combinatory therapy. Excitingly, this could prove to be a promising avenue for recovery after SCI (Table 4). Activation of cAMP signalling by rolipram and further cAMP injections combined with Schwann cells transplantation to the injury site following spinal cord contusion in rats further increased axonal sparing, myelination and locomotor function compared to the individual components (Pearse *et al,* 2004b). It was apparent that rolipram was responsible for most of the beneficial effects and the transplants alone had a minimal effect (only significant improvements to myelination). This suggested that growth factors might be required to promote axonal growth through the graft. Indeed, rolipram combined with the further addition of modified Schwann cells to secrete a bifunctional neurotrophic molecule (D15A) with both NT‐3 and BDNF activity increased numbers of axons originating from the reticular formation below the lesion/implant area and provided the greatest improvement in hindlimb function (Flora *et* *al,* 2013). Similar to the previous study, rolipram was the main contributor to the beneficial effects compared to a minor improvement observed by BDNF + NT‐3. This indicates that activating the intrinsic regenerative programme is an approach that may be superior to cell‐ or growth factor‐based therapies (Table 4).

Microtubule stabilisation is fast becoming an attractive therapeutic option for promoting intrinsic regenerative capacity as well as limiting the deposition of inhibitory molecules in the injured CNS. Recently, enhancing and directing the growth of docetaxel‐stabilised microtubules by the co‐treatment of the growth factors BDNF and aFGF in liposome hydrogels in a rat thoracic contusion injury model was achieved (Wang *et* *al,* 2018). This application supported regeneration by improving neuronal survival and plasticity, and rendering a more permissive extracellular matrix environment, leading to significantly improvement of BBB score points compared to untreated controls (Wang *et al,* 2018). Co‐administration of taxol with the EGFR signalling antagonist cetuximab verified therapeutic effects towards inhibition of scar deposition and promotion of neuronal differentiation, axonal outgrowth and functional recovery in a severe SCI model in rats (Table 4; Fan *et* *al,* 2018). The precise role of cetuximab in this scenario is unclear, although it was demonstrated to promote the differentiation of injury‐activated endogenous‐NSC differentiation into neurons. There is clearly need for more investigations to continue in this area of research. The CRMP family of proteins regulate aspects of neurite growth through stabilising microtubules and phosphorylation of CRMP2 by GSK3β reduces CRMP2's binding affinity for tubulin heterodimers leading to microtubule depolymerisation (Fukata *et* *al,* 2002). Interestingly, preventing CRMP2 phosphorylation by pharmacological inhibition of GSK3β was found to be a critical mediator of the neurotrophic response in DRG neurite outgrowth (Nagai *et al,* 2016). This suggests that targeting actin dynamics may also potentiate the efficacy of neurotrophic delivery which could be an attractive strategy.

### Combinations of therapies with rehabilitation

Despite the many efforts made by patients, physiotherapists and trial designers, rehabilitation as a treatment for SCI achieves a modest recovery at best. Could combining rehabilitation with therapies that aim to restore intrinsic growth capacity and/or remove inhibitory factors be more effective? Plasticity‐promoting therapies including ChABC can restore neuroplasticity to a level it appears in early childhood and promote compensatory sprouting of spared fibres to form new neural connections. Yet, there is frequently a mismatch between anatomical and functional recovery. For example, treatment of rat cervical spinal cord injuries with ChABC produced a modest recovery in CST function, as measured by skilled paw function despite a large observable increase in axonal regeneration and neuronal plasticity (Garcia‐Alias *et* *al,* 2008). Are new neuronal connections ineffective if the host does not know how to use them? Promoting plasticity by itself may not be sufficient to promote functional recovery if it leads to random new connections. The formation of appropriate connections in the spinal cord and brain may need to be driven by appropriate rehabilitation. Indeed, subsequent studies have shown that ChABC treatment combined with rehabilitation leads to enhanced recovery through opening a window during which rehabilitation becomes more effective in the newly plastic environment (Fawcett & Curt, 2009; Garcia‐Alias *et al,* 2009; Wang *et al,* 2011a; Shinozaki *et* *al,* 2016). Therefore, utilising appropriate rehabilitation regimes is emerging as a promising avenue for shaping the new circuits created by therapies into useful new connections (Table 5). However, the timing of the treatment and rehabilitation appears to be critical. In one experiment involving thoracic spinal cord contusion in rats, the animals developed an abnormal stepping pattern when rehabilitation was included in parallel with the treatment of anti‐Nogo‐A antibody (Maier *et al,* 2009). Similarly, synchronous treatment of anti‐Nogo‐A antibody with rehabilitation in a model of stroke resulted in “chaotic hyperinnervation” leading the researchers to hypothesise that rehabilitation at an early stage was reinforcing erroneous connections (Wahl *et* *al,* 2014). Instead, sequential rehabilitation regimes are astonishingly effective and in some cases can almost completely restore specific motor tasks (Table 5; Garcia‐Alias *et al,* 2009; Chen *et* *al,* 2017). More recently, endogenous CSPG degradation by an astrocyte‐selective AAV‐ADAMTS4 gene therapy combined with hindlimb rehabilitation enhanced functional recovery following rat thoracic SCI compared to either intervention alone (Griffin *et* *al,* 2020).

Current reports for the role of rehabilitation with cell transplantation are limited to one preclinical study which enlisted autologous bone marrow stem cells with swim training where a difference to functional parameters between groups was observed (Carvalho *et* *al,* 2008). As we discussed above, considering swim training is less effective than high‐intensity quadrupedal training, it would be of interest to test cellular transplantation with other regimes of rehabilitation. Rather, combining rehabilitation with cell transplants in human has progressed immediately to trials (Table 6). These trials have demonstrated that cell transplantation into the injury site seems to be safe and appears to only show beneficial effects for patients when combined with rehabilitation further highlighting the importance of combinatory approaches in the clinical setting (Li *et* *al,* 2015; Zhu *et* *al,* 2016; Anderson *et* *al,* 2017). Similarly, current reports for the role of rehabilitation with neurotrophic factors are limited to two preclinical studies. First, acute AAV‐mediated expression of NT‐3 combined with sequential spinal electromagnetic stimulation rehabilitation, swim training and exercise ball training improved electrophysiology recordings, narrow beam task, error ladder task and several Catwalk gait analysis parameters (Table 5; Petrosyan *et* *al,* 2015). This result was only observed with the combination of electrical stimulation plus exercise or electrical stimulation plus exercise plus AAV‐NT3, whereas any singular therapy had no effect. This indicates that electrical activity in the spinal cord may be a requirement for activity‐induced neuroplasticity. Second, intrathecal administration of ChABC, PDGF, bFGF and EGF combined with daily quadrupedal treadmill training leads to enhanced neuroanatomical plasticity of CST and the serotonergic‐spinal pathway, although these changes failed to translate to functional improvements (Alluin *et al,* 2014). Considering there are many variables between these two studies, at the stage it is difficult to draw larger conclusions.

**Table 6:** Current published clinical trials for spinal cord injury

Name of therapy	Mechanism	References	Key findings	Current status of trials
Methylprednisolone	Neuroprotection: Anti‐inflammatory corticosteroid	Bracken *et* *al* (1984, 1990, 1997), Matsumoto *et* *al* (2001)	Ultimately, no convincing improvement to motor and sensory functions. May lead infection, pulmonary, gastrointestinal complications	Completed Clinical use in some countries but not all
GM‐1 ganglioside (Sygen)	Neuroprotection and regenerative properties	Geisler *et* *al* (1991, 2001)	No statistical significance between trial groups achieved	Completed Rarely used in the clinical setting
Thyrotropin‐releasing hormone (TRH)	Neuroprotection: Prevents apoptosis	Pitts *et* *al* (1995)	No validity for clinical use	Completed No current clinical use
Nimodipine	Neuroprotection: Calcium‐channel blocker	Pointillart *et* *al* (2000)	No significant difference among all groups in the trial	Completed No current clinical use
GK‐11 (gacyclidine)	Neuroprotection: NMDA receptor antagonist	Tadie (2003)	No statistical significance between trial groups achieved	Phase II completed No current clinical use
Transplantation of peripheral nerve graft and aFGF–fibrin glue	Tissue replacement: Repair/regeneration	Cheng *et al* (2004), Wu *et* *al* (2008, 2011)	Treatment is well tolerated and safe Significant improvement in ASIA scores Impetus for a phase III trial	Three phase I/II trials completed No current clinical use
Transplantation of autologous activated macrophages	Tissue replacement: Repair/regeneration	Knoller *et* *al* (2005)	Appears to be safe. Three patients improved ASIA score	Phase I completed Phase II suspended for financial reasons
Human adipose tissue‐derived mesenchymal stem cells	Tissue replacement: Repair/regeneration	Ra *et* *al* (2011)	Safe and tolerable; Impetus for subsequent trials	Phase I completed Phase II trials underway Further trials underway
Cethrin (BA‐210; SPRING‐VX‐210)	Repair/regeneration: RhoA blocker	Fehlings *et al* (2011)	Safe and tolerable Improved ASIA motor scores in cervical SCI patients Impetus for subsequent trial	Phase I/IIa completed Phase IIb/III underway No current clinical use
Minocycline	Neuroprotection: Tetracycline antibiotic	Casha *et al* (2012)	No statistical significance between trial groups achieved	Phase II completed Phase III RCT ongoing No current clinical use
Granulocyte‐stimulating factor	Neuroprotection	Takahashi *et* *al* (2012)	Safe and tolerable ASIA score increased by one point in 9 of 16 patients	Phase I/IIa Completed No current clinical use
Transplantation of autologous Schwann cells	Tissue replacement: Repair/regeneration	Review of 2 trials: Guest *et* *al* (2013) Anderson *et al* (2017)	Initial trial reports from Iran and China suggest clinical safety Recovery of function motor and sensory function in many patients although more characterisation is needed	Phase I completed No current clinical use
Riluzole	Neuroprotection: Sodium‐channel blocker	Grossman *et* *al* (2014)	Safe and tolerable. Improved ASIA motor score Impetus for subsequent trial	Phase I completed Phase IIB/III trials discontinued No current clinical use
Umbilical cord mesenchymal stem cell transplantation	Tissue replacement: Repair/regeneration	Cheng *et* *al* (2014) Zhu *et al* (2016)	Significant improvements in some patients	Phase II completed
Olfactory ensheathing cells	Tissue replacement: Repair/regeneration	Meta‐analysis of eleven articles: Li *et al* (2015)	OEC transplantation appears to be safe Evidence for efficacy is modest Further RCTs required to determine benefit	Trials completed in Portugal, China and Australia Patients can pay for treatment in Portugal and China
Autologous mesenchymal stem cells	Tissue replacement: Repair/regeneration	Oh *et* *al* (2016)	Safe but has a very weak therapeutic effect	Phase III clinical Trial completed
Autologous bone marrow stem cell transplantation	Tissue replacement: Repair/regeneration	Satti *et* *al* (2016)	Safe and tolerable	Phase I completed
HuCNS‐SC (human foetal neural stem cells)	Tissue replacement: Repair/Regeneration	Levi *et* *al* (2018)	No safety concerns	Phase I/II for thoracic and cervical SCI complete
ATI‐355 (anti‐Nogo‐A antibody)	Repair/regeneration: Blocks inhibitory Nogo	Kucher *et al* (2018)	Safe and tolerable Impetus for subsequent trial	Phase I complete Phase II underway No current clinical use

Rehabilitation is limited in complete injuries, yet considering neuronal networks below the lesion site remain active, combining treadmill rehabilitation with neurotransmitter administration and electrical modulations in the lumbar spinal cord can lead to the re‐establishment of walking ability in completely transected rats, a major step towards future therapy for completely paralysed patients (van den Brand *et al,* 2012). The use of electrical stimulation with rehabilitation in humans has been practised in the clinic for many decades. This can be achieved by functional electrical stimulation (FES; on the skin), epidural electrical stimulation (EES; stimulation of the spinal cord directly). FES has recently made large strides towards helping people with SCI. A major breakthrough showed that EES in combination with intense rehabilitation and neurotransmitter administration could help to restore walking function in three individuals with varying levels of incomplete SCIs (Wagner *et* *al,* 2018). This may be the result of the precise timing of the stimulation rather than continuous stimulation which may facilitate the naturally occurring signals. However, this study did not include a group of rehabilitation without stimulation and it is possible that the high‐intensity orthotic‐assisted rehabilitation programme is the main therapeutic component.

## Towards clinical translation: current status

Many therapeutic strategies have been discussed in terms of their potential to address the human condition since their conception, yet relatively few SCI clinical trials have been completed (Table 6). For those that have reached clinical trials, none have reached general clinical application, aside from some countries that offer medical tourism for non‐validated therapies. Trials to overcome the devastating effects of SCI can be categorised into neuroprotective and regenerative approaches. Unfortunately, neuroprotection trials for human SCI have largely failed (Kim *et* *al,* 2017; Santamaria A.J., 2017). For example, trials from the neuroprotective interventions methylprednisolone, GM‐1 and gacyclidine failed to show sufficient statistical evidence for efficacy to be approved for clinical use (Table 6). Neuroregeneration/repair trials have emerged more recently, yet only a small number of treatments have reached the stage of human testing. These include anti‐Nogo‐A antibody therapy and Cethrin, and various cell‐based therapies, which have each completed phase I/IIa trials with no apparent adverse effects and some indication of functional improvements (Fehlings *et al,* 2011; Kucher *et al,* 2018). Trials of cell transplant therapies remain controversial in terms of ethical, safety and efficacy, but there is, at least, evidence that transplants may improve some sensory/motor and bladder functions (Fan *et* *al,* 2017). An exciting future prospect is for the human application of ChABC, which is yet to reach clinical trial; however, the evidence from the recent trials of SCI in dogs and rhesus monkeys supports ongoing preclinical efforts (Hu *et al,* 2018; Rosenzweig *et al,* 2019). The drive towards translating the promising results of ChABC to being clinically available is currently led by a collective team named the CHASE‐IT (ChABC for Spinal Injury Therapy) consortium. A focus of the consortium is now optimising the LV‐ChABC gene therapy approach to making it more clinically safe by using an AAV vector instead and with gene regulation (International Spinal Research Trust WEBARTICLE, 2019). Such approach would allow for robust, prolonged and controllable expression of mChABC in the human spinal cord which may exceed the efficacy achieved by infusion of the native enzyme. We are on the cusp of observing auspicious therapeutic candidates entering human trials; however, the road towards their clinical translation is arduous, and many factors need to be considered.

### Problems for translation

One major problem towards clinical translation is the lack of robustness and reproducibility in preclinical studies (Schaffran & Bradke, 2019). With this notion, the “Facilities of Research Excellence‐Spinal Cord Injury” (FORE‐SCI) programme was developed to replicate key studies in the SCI field (Steward *et* *al,* 2012). Out of 12 studies selected to be replicated, it is perhaps worrying that only one study was fully replicated, six were not and the rest had mixed results or were only partially replicated. Therefore, it is imperative that a confirmatory replication of key aspects of the original study by an independent laboratory is conducted before the therapy progresses to the clinical stage. In academic research however, replication studies are rarely performed due to a lack of funding, the absence of recognition for performing such studies or problems with publishing. This is something that needs to change for us as a community to progress. This will further become more complicated when multiple strategies are combined.

Combinatorial approaches are likely to require tailored experimental design and analytical frameworks. The complexity of the combinatory treatments may project deceivingly incremental increases in efficacy. It is difficult to discern the involvement of the individual treatment entity when there are many variables that plague the reproducibility between studies and between laboratories. Facing a slew of possible therapies and combinations of such, it is a good idea to wait until we have more data before trials are initiated. What is the best cell type and biomaterial for tissue replacement? What are the ethical concerns or potential for harm when obtaining these cells? What growth factor or combination of growth factors is the best? What is the best method for removing inhibitory factors? Is the removal of multiple types of inhibitory factors required? Is the experimental model relevant to the human condition that the therapy is intended for? The experimental model should as closely represent the human condition, fine hemisection manipulations, for example, do not. Contusion and compression injuries are certainly more clinically relevant, though this does not make them the best choice for every experiment. Another consideration is that the spinal cord of humans is much thicker, has different anatomy, and drug penetration is very different. It would be preferential for therapies to be tested in multiple models, ideally including a larger animal (primate or porcine), to broaden our understanding of the limitations of the treatment. Another major problem is that outcome measures of motor improvements in humans are relatively insensitive compared to animal behavioural tests. The ASIA impairment scale is outdated, and a 5‐point scale ranging from full movement to no movement is underperforming. However, FORE‐SCI groups are developing additional tests of sensory and motor function that should allow more sensitive testing of outcomes after SCI in clinical trials. Another important consideration is that non‐motor system directly affects quality of life and often out‐ranks in importance to patients (Simpson *et* *al,* 2012). Often multiple aspects of health or motor functions are not reported in studies and so we would not see these kinds of side effects that could manifest in humans. Careful analysis of alterations in not only the locomotor system but also sensory, autonomic and possibly cognitive function should also be assessed in relevant animal models prior to entering human clinical trials. The complexity of clinical trial designs featuring combination therapies will also be an important factor to take into consideration during therapeutic translation (Fawcett *et al,* 2007). Ultimately, preclinical researchers and trial designers need to communicate better and more often. Trials must be large enough to prove efficacy, reflecting the parameters and outcomes of the preclinical studies.

## Conclusions

SCI results in many problems that need to be addressed in order to discover a cure for the condition. Many promising individual therapies have been developed and are progressing towards clinical trials. However, many *in vivo* studies fail to be replicated and often fail to translate clinically which is unacceptable to individuals with SCI anxiously awaiting therapeutic options. The reproducibility of results from promising candidates must be assured before progressing to human clinical trials. Many combinational strategies have demonstrated greater beneficial outcomes than the individual components alone by addressing multiple aspects of the pathology, but these need further characterisation to ensure meaningful efficacy beyond their individual components. Clinical trials in the future will eventually need to focus on combining the best therapies that aim to address as many of the problems as possible. The scenario will become increasingly complex as this happens and conversation between basic researchers and clinicians must happen to ensure accurate study design and functional readouts.

**Pending issues**

(i) What is the ideal combination of treatments?

(ii) How to assure the reproducibility of preclinical research?

(iii) What is the efficacy of combinatory treatments in larger animal models?

(iv) Are these feasible, safe and efficient for clinical translation?

(v) How to proceed with clinical trials involving combinatory therapies?

## Conflict of interest

H. Witte, A. Ertürk, F. Hellal and F. Bradke filed a patent on the use of microtubule‐stabilising compounds for the treatment of lesion of CNS axons (European Patent no. 1858498; European patent application EP 11 00 9155.0; U.S. patent application 11/908,118). The authors declare no competing financial interests.

## For more information

(i) https://www.spinal-research.org/
(ii) https://www.christopherreeve.org/research
(iii) https://www.wingsforlife.com/de/
(iv) https://scicrunch.org/odc-sci
(v) https://asia-spinalinjury.org/
(vi) https://www.clinicaltrials.gov/ct2/
(vii) https://www.neuron-eranet.eu/
(viii) https://www.themiamiproject.org/
